# The Lived Experiences of Patients with Head and Neck Cancer during Concurrent Chemoradiation Therapy Care Process

**DOI:** 10.31557/APJCP.2020.21.12.3669

**Published:** 2020-12

**Authors:** Sirikorn Kongwattanakul, Pranom Othaganont, Wen-Chii Tzeng

**Affiliations:** 1 *Faculty of Nursing, Thammasat University, Prathum Thani, Thailand. *; 2 *School of Nursing, National Defense Medical Center Neihu District, Taip, Taiwan. *

**Keywords:** Concurrent chemoradiation therapy, patients with head and neck cancer, care process, radiation oncology

## Abstract

**Purpose:**

An exploration of concurrent chemoradiation therapy care process from the perspective of patients with head and neck cancer can provide an insight to their lived experience and the difficulties they encounter in daily life towards a deeper understanding of this phenomenon to shape nursing service delivery. The aims of this study were to explore the lived experiences of patients with head and neck cancer while receiving concurrent chemoradiation therapy.

**Methods:**

Data were generated from individual in-depth interviews with fifteen head and neck cancer patients, according to the semi-structured interview guidelines, at the out-patient radiation oncology department, Chulabhorn Cancer Center, Bangkok, Thailand.

**Results:**

By using Graneheim and Lundman’s content analysis, three categories from the data analysis of patients with head and neck cancer receiving concurrent chemoradiation therapy were isolated: 1) overwhelming information, 2) unpleasant symptom cluster, and 3) strategy for adherence to treatment regimen.

**Conclusion:**

The findings help to provide a better understanding of the lived experiences of patients with head and neck cancer during concurrent chemoradiation therapy, in terms of their suffering from various unpleasant side effects and how these impact their life along the treatment journey. This perspective on the care process in these patients enhances the development of a nursing care model based on patient-centered care toward positive patient outcomes.

## Introduction

For locally advanced head and neck cancer, multimodal therapeutic strategies, including surgery and adjuvant radiation or chemoradiation, are the current standards of treatment depending on the pathologic features of tumor (Conley, 2006). Despite the main effective treatment for advanced stage head and neck cancer, concurrent chemoradiation therapy (CCRT) leads to multiple symptoms and affects a patient’s quality of life (Fang et al., 2007). A high rate of severe toxicity from CCRT has been reported in patients receiving CCRT (Fang et al., 2007). Disease and treatment-related symptoms can be particularly debilitating and patients can suffer from both acute- and late-side effects of physical, functional, and psychosocial problems (Wells et al., 2015).

 To date, many research studies have focused on the assessment of advanced techniques, with the efficacy of treatment modalities, and management of side effects and toxicities. Whereas, only a few studies have been conducted to understand patients’ experiences and management of their head and neck cancer (HNC). Being diagnosed with cancer may cause major distress for HNC patients (Fitchettet al., 2018). Whilst, many negative thoughts and the suffering of individuals with HNC include: 1) shock, anxiety, and fear of dying while attempting to make sense of their diagnosis, 2) distress from disrupted expectations and changes in daily and life routines, 3) rising awareness of self, others, and healthcare systems, 4) usage of strategies to tolerate treatment and manage changes, and 5) living with a dubious future (McQuestion and Fitch, 2016). These mental and psychological burdens may present unique problems for an individual’s well-being, sense of control, social functioning, and quality of life (Stuiver et al., 2008). Disruptions in eating, swallowing, and speaking are central to social functioning and particularly during treatment. Whereas, the side effects of skin changes can produce high incidences of anxiety and depression (McQuestion et al., 2011). There have been many evidences to support the notion that the effects of radiation therapy on treated areas lead to tissue damages and local symptoms, depending on physiology and organ functions. 

In providing effective care, the establishment of patients’ experiences in the care process is crucial. The patient’s perspectives on the radiation therapy care process can provide some insights to their experiences during the nursing service delivery. The roles of radiation oncology nursing to enhance patients and their family’s involvement during the complicated process of treatment and assist them in dealing with treatment-related side effects are challenging. Accordingly, radiation oncology nurses engaging in the management of patients’ treatment-related symptoms and toxicities should identify the parameters for particular situations which lead to the non-adherence. Hence, this study purposed to explore HNC patients’ lived experiences during the care process of concurrent chemoradiation therapy.

## Materials and Methods

*Study design*

A descriptive qualitative design study was performed to investigate the perspectives of HNC patients over a four-week period, with semi-structured interviews for data collection. Data were then analyzed using Graneheim and Lundman’s qualitative content analysis (Graneheim and Lundman, 2004).

*Interviews*

In order to gain a deeper understanding of patients’ lived experiences and any side effects during the combined radiation therapy with chemotherapy and their needs throughout the care process, the semi-structured interviews were performed with the approval of five experts. The interviews were tape-recorded, hand-written, and transcribed verbatim. 

*Participants and setting*

A number of 15 outpatients who underwent different phases of concurrent chemoradiation therapy process at Department of Radiation Oncology, Chulaborn Hospital, Thailand, for a daily course of treatment up to seven weeks were approached to participate in this study by the principle researcher at the outpatient clinic (Table 1). Every participant who was eligible at the study period was invited to participate. Participants were eligible if age over 18 years and receiving the combination of radiotherapy and chemotherapy with curative intent, ability to communicate in the Thai language, and willing to participate in this study. Written informed consent forms were signed and obtained from all participants before the interviews. Participants were excluded if they significantly had severe co-morbidities or another illness required for hospitalization (such as dyspnea, SVC obstruction, and sepsis), which could obstruct the interview and may impair participation in caring process and research process, or incurable cancer or palliative radiation therapy as defined by radiation oncologist (such as metastasis cancer and end of life stage cancer). 

Those diagnosed with head and neck cancer included nasopharyngeal cancer (n=9) and non-nasopharyngeal cancer (n=6). All of them received the concurrent chemoradiation therapy (n= 15). Of these, there were four women and eleven men, with age ranged between 31 and 81 years (average age of 47 years). The patients’ interviews were done in different phases, including during treatment (n=7) and within 6 months after completion of treatment (n= 8). 

The CCRT process started with patients’ consultation in preparing to receive radiation therapy (RT) and CT-simulation with positioning and planning for radiation doses. The radiation treatment (ranging from 5-7 weeks on average) included weekly monitoring of any side effects by the radiation oncologists and radiation oncology nurses, with follow-up for any complications and disease recurrence. The commonly used chemotherapy regimen was scheduled every three weeks with 100 mg/m2 and high-dose bolus. The patients normally required to stay in hospital for a few days.

*Analysis*

To synthesize and summarize patients’ lived experiences, the inductive content analysis by Graneheim and Lundman was employed with levels of concepts, including latent content, unit of analysis, meaningful units, condensing, abstracting, content area, code, category, and theme (Graneheim and Lundman, 2004). The audio-recorded interviews were transcribed and data were analyzed thematically. Data from interviews, audio recordings, and peer reviews were integrated to ensure the trustworthiness of all information. This study applied the triangulation together with co-researcher and participants in checking the findings. The confidentiality and anonymity of participants’ information was ensured through data management.

Rigor: The principle of trustworthiness suggested by Guba and Lincoln (1989) was applied to ensure the rigor of the study as much as possible (Guba and Lincoln, 1989). The four qualitative trustworthiness criteria included credibility, transferability, dependability, and conformability with specific strategies throughout the research process (Krefting, 1991).

## Results

The participants’ characteristics were presented in Table 1. 

*Category 1: Overwhelming information*

According to the interviews, the majority of participants needed information about the plan, cost, and length of treatment, as well as self-care and self-preparation for the planned treatment. Some of the patients did not even comprehend what they should know or ask, especially the elderly people. These patients let their family members manage their routine activities and did not want to know the stage of their disease. 

“*I do not think too much. What the doctor says is what I should do and I do meditate everyday*” P001 (75 yo), “*Nothing has changed; whatever will be, will be. I am not afraid.*” P009 (76yo)

In contrast, the middle-aged cancer patients had a list of questions in hand and participated more in their care process. Sometimes, they came with their own reviews or contents from medical journals to discuss with doctors and nurses. 

“*I was searching tons of information on the Internet, and I am afraid that I cannot complete the course of treatment as prescribed and also I am worrying about having a tube in my stomach.*” P003 (31 yo) 

They asked many questions about the information that they could not find in booklets from their care providers at the clinic. Nonetheless, the patients’ concerns and their understanding of the information were not very much taken into consideration. If they did not asked any questions, it was assumed that they understood all of the information given.

*Seeking tailored information*

During the RT process from week 1 to week 4, the patients and their family caregivers sought information about the self-care and the adherence to treatment course. They were provided with important information by radiation treatment providers at the beginning of treatment process. 

“*I have searched for information about cancer treatment but what I heard from the doctor makes me panic.*” p 018

Numerous booklets and leaflets were also given by doctors, nurses, and radiation therapists on the first day to take home and read to make sure that they understood everything in the care process. 

“*I did not know what to ask the doctor or the nurse when I first came here. They gave me a lot of information. The only thing I know is I want to be cured.*” p015

Talking with healthcare providers was the main channel for obtaining the information they needed. Informal talks with others patients made some of the participants more frustrated. 

“*Talking with other cancer patients… makes me panic more, and sometimes it makes me feel better to have someone to talk to. So, I decided to talk with no one.*”P017

*Chemotherapy information provision*

During the treatment, patients had to face new experiences. They could ask for assistance from doctors and nurses to handle with any side effects. Significantly, most of them disclosed that they did not receive information about chemotherapy or self-care. 

Chemotherapy was not given on the same day at the end of the radiation therapy schedule or may be delayed a week after completing the radiation therapy. Some patients were ordered the adjuvant chemotherapy after the completion of radiation treatment. The regimens may cause patients to hesitate for the continuity of chemotherapy, especially in those with unfavorable experiences of severe side effects.

“*I want to have information about both because I have to receive concurrent chemo-radiation therapy.*”p018

*Category 2: Unpleasant symptom clusters*

The combination of radiation therapy side effects and toxicity of chemotherapy contributed to the co-occurrence of both local and systematic symptoms, causing patients’ physical and psychological distress. In all patients, three or more concurrent symptoms were related to one other and may or may not share the same etiology.

*Physical symptoms*

From the beginning, some of the patients did not have any symptoms, but during the process they had to make sense of severe toxicity, particularly when radiation was combined with chemotherapy. Nonetheless, the treatment schedule could be changed depending on their health status. Almost all of the participants rarely presented with a single symptom. They encountered multiple symptoms and each individual rated their suffering differently. 

The consequences of active treatments disrupted the patients’ quality of life with intense symptoms. The combined dosages of radiation treatment also destroyed both the mucosa in the oral cavity and the salivary gland, leading to the effects on dietary pattern, decisions regarding food intake, and difficulty in chewing and swallowing, which then caused poor health status. 

“*Not being able to eat anything was the worse symptoms for me during treatment. Chemo makes me felt nauseated and I vomited and I also did not to eat.*”P010

*Psychological symptoms*

During the visit to Department of Radiation Oncology Department, the patients and their family caregivers always came with hope, but at the same time they had anxieties about the treatment process, side effects from radiation with or without chemotherapy, and fear of radiation therapy. They expressed their fear of living close to their love ones because of radiation exposure.

“*The big machines and strange immobilization devices are scary.*” P009

 “*Why is it so painful? Why do I have to suffer from the radiation and chemotherapy treatment so much? I cannot sleep and I cry until have no tears left.*” P010

At the first day of radiation treatment, the patients changed from the CT-simulation room to the actual treatment room, which was in a different location. Patients had to experience the new machine and stayed alone in the treatment room, which made them fearful.

“*In the simulation room, and treatment room, there was so big machine and it was noisy. It was annoying me and that prevented me from staying still.*”P009

*Category 3: Strategies for adhering to treatment regimen*

The patients suffered much more from toxicity and other side effects than radiation alone. Even though they had to face the same situation, radiation therapy gave them different experiences. They required support and symptom management.

*Support*

Cancer patients individually took the initiative care or received assistance from their family caregivers with recommendations and supports from radiation specialists. The radiation treatment took 6-7 weeks (5 days a week). The flows of radiation schedule and service were eventually familiar to patients. They got the radiation time slot two days in advance and received the same screening every day, such as registering, having their vital signs taken, being billed for the cost of treatment, and contacting the cashier. This main information was provided by radiation oncologists, radiation therapists, and radiation oncology nurses in the department as a routine.

“*After talking with the doctor, it made me trust her. The nurses give me information as a friend and do not treat me like an inferior.*”P004

Support from family and friends, as well as the radiation oncology team, had the most positive impact on patients during treatment course. 

Despite their suffering from treatment toxicity, the cheerful words or the company of their family caregivers created motivation for them towards treatment adherence. 

“*The staff here is very kind and they talk to me as though I am a relative.*” P001

*Symptom management*

It was crucial for proper management of any symptoms and side effects from treatment in a bid to enhance treatment adherence. During the radiation treatment, patients met with radiation oncologists and radiation oncology nurses at least once a week. They may ask the healthcare providers about their concerns related to the side effects of radiation. They received their supportive care, such as medication in case of any pain or skin reaction. Some patients tried to add more nutrients to their diets for energy replacement. Some patients could maintain their health status for many weeks, but others may feel nothing or get worse, such as from skin reaction. 

Patients sought new and different strategies to alleviate their symptoms and side effects from informal channels, such as other cancer patients or healthcare professionals. Some patients utilized food recipes to maintain their appetite. Others used meditation, praying, and reading books before going inside the treatment room to calm themselves. After the completion of radiation treatment, the patients received an appointment with doctors for approximately 4 weeks later for follow-up on any acute side effects and treatment outcomes. 

“*I have imagined what I have eaten before and what it tasted like to allow me to eat more food. I cannot eat spicy food. I do not want to count the days of treatment I just let it go day by day.*” P 018

*Fight or flight*

The course of treatment took 6 to 7 weeks for one RT regimen. Mostly, the symptom of distress escalated during week 3 and worsened through the entire course. The last two weeks of the concurrent chemoradiation process was the toughest time for patients and their family caregivers. The patients could adhere to treatment. However, the patients felt tired and discouraged when the same symptoms occurred, then they could not tolerate the treatment any longer.

This was the time that patients needed the most support and cheerful encouragement from the healthcare team and their families. The idea of fight or flight from the treatment process grew with the peak of toxicities. Mouth pain, plus skin wounds from treatment, caused the most suffering. Some patients dropped out, or strayed from their course of treatments due to these side effects. A chemotherapy regimen may be given to patients after completing their radiation therapy, following the unpredicted symptom experiences and the importance of the continuity of treatment. 

“*I do not know what will happen next. Now I am at the 15*^th^* radiation treatment. Sometimes, I pray before going into the treatment room.*” P017

“*The worse experience…was the positioning with the immobilization mask. It was terrible. I could not breathe normally. Every day before I came to receive radiation I had to pray but eventually some days I could not make it.*” p001

The categories and codes of patients’ lived experiences concerning the radiation treatment process were presented in Table 2. 

**Table 1:** Patients’ Demographic and Medical Characteristics

Demographics	Data/status	n = 15
Gender	Male	11
	Female	4
Age	31-45	3
	46-60	7
	60-80	4
	>80	1
Education	Less than a college degree	6
	College degree or higher	9
Occupation	Yes	13
	No	2
Marital status	Married	13
	Other	2
Family caregiver	Yes	15
		
Patient payment	Government employee	2
	Self-paid	4
	Universal coverage	7
	Security insurance	2
Comorbidities	No	11
	Yes	4
Religion	Buddhist	15
Surgery	No	6
	Yes	9
Feeding tube	No	9
	Yes	3
Site of head and neck cancer	Non-nasopharyngeal cancer	6
	Nasopharyngeal cancer	9
Radiation treatment	Concurrent chemoradiation	15
Number of RT fractions	33	15
Chemotherapy	Cisplatin	14
	Carboplatin	1

**Table 2:** The Categories and Codes of Patients’ Lived Experiences on Radiation Treatment Process

Code	Sub-categories	Category
Too much information on day 1 Cannot remember Confusion What I need to know Searching from website	Overwhelming information	Seeking tailored information
I know nothing Lack of information about chemotherapy	Chemotherapy information provision	
Sore mouth, sore throat, thick saliva, dry mouth, difficulty swallowing Taste change, weight loss Skin burn Nausea, vomiting Fatigue, dizziness, drowsiness Bloating, constipation, GERT Blood, kidney malfunction	Physical symptoms	Unpleasant symptom cluster
Mask tight Worry Fear Suffering Hopeless	Psychological symptoms	
Make sense of the situation Talk with others Support from friends Support from family Support from doctor, nurse, Therapist Good environment, atmosphere	Support	Strategies to adhere to treatment regimen
Praying Tips from others Asking experts Reading Meditation Family support	Symptom management	
Wait and see Trial and error	Fight or flight	

## Discussion

In this study, HNC patients’ experiences of the concurrent chemoradiation therapy revolved around three key concepts: 1) overwhelming information, 2) unpleasant symptom cluster, and 3) strategy for adherence to treatment regimen.

Radiation therapy is the cause of fear and anxiety, while patients with cancer think that this type of treatment is a mysterious aspect in their life. Moreover, radiation therapy-related toxicities are unknown and they have to seek more information. The combination of chemotherapy and radiation therapy can cause more side effects than just radiation alone. This combination of treatment can develop the most severe symptoms among patients. Treatment adherence and person-centered care concepts are critical to improve treatment outcomes, including patient participation, which should be the main concern of the care system towards good clinical outcomes (Delaney, 2018; Rehaman, 2018).

In radiation therapy setting, specific information is rarely provided to the general public, so patients will search for information from websites, or ask their family and friends, who are often not able to provide relevant, meaningful information or answers. Preparing the patients with head and neck cancer for what to expect over the course of treatment has to be individually tailored in accordance with each individual’s personal learning styles and preferences. In a previous study, the tailored information with effective communication could support the nursing care towards the relief of treatment-related symptoms and some distress in patients with head and neck cancer (Rojthamarat, 2018).

The experiences of unfavorable symptoms during the concurrent chemoradiation treatment can occur from the first week and peak during the third week, including alterations in tasting, pain in the mouth, loss of appetite, loss of saliva, burns to the skin, thickening of saliva, and fatigue (Hollander-Mieritz et al., 2019). Symptom management in head and neck cancer cases would suggest only supportive treatment, such as analgesics, anesthesia spray, or a feeding tube to maintain nutritional status. To classify or identify the symptoms in a cluster would be beneficial for healthcare providers in management of important symptoms and evaluation of the status outcomes of symptoms for effective administration. The severity of symptoms do not usually decreases, but gets worse even when treatment is accomplished (Rosenthal et al., 2014).

Currently, multiple symptom management and involvement of patients in the identification of symptom clusters are beneficial across symptoms to improve clinical outcomes (Kwekkeboom, 2016). Information and education from healthcare providers as a guidance of symptom management is necessary, but at the same time patients have to also adjust their daily life routine (Rojthamarat, 2018). Therefore, the understanding of symptom cluster burdens in patients with head and neck cancer at the time of treatment initiation is crucial in the radiation therapy care process. Not only the oriented physical problems but also the psychological symptoms are essential for management and improvement of the quality of life along the treatment continuity (Hanna et al., 2015).

Cheerful speech, understanding patient’s experiences, concerns, and preferences for information, as well as language, financial issues, transportation, and other supports from the radiation oncology nurses are very important for helping patients to overcome their suffering before, during, and after treatment. Family and social supports are key factors to encourage patients during treatment, and especially in their routines, such as cooking, accompanying them to clinic, cheerful speech from family member, etc. (Baenziger et al., 2015). In the meantime, some patients could stay calm and positive by praying, reading, and information seeking from doctors and nurses.

Advanced technology with large unknown machines (to the average patients) exacerbates anxieties about the radiation process in patients (Rose and Yates, 2015). The treatment course of radiation therapy takes almost two months, together with chemotherapy, which can cause suffering to patients (Pan et al., 2017). Fight or flight ideas from patients can occur throughout the course of treatment. Uncertainty is also associated with the severity of symptoms from side effects and toxicities during treatment. Hence, these results provide many challenges in nursing care for patients with head and neck cancer throughout the treatment course. 

In conclusion, most patients with head and neck cancer experience their suffering from many unpleasant side effects which impact their quality of life along the treatment course of concurrent chemoradiation therapy. The study results provide nurses and other healthcare professional with a deeper understanding of patients’ experiences as they face the same situation, but see things differently and make different decisions. The development of a nursing care model based on patient-centered care is thus recommended to enhance favorable treatment outcomes and positive patient satisfaction. 
